# The Impact of Preoperative Risk Factors on Peritoneal Dialysis-Related Peritonitis: A Single-Center Prospective Study in Japan

**DOI:** 10.3390/medicina58020313

**Published:** 2022-02-18

**Authors:** Toshihiro Sato, Go Anan, Takuo Hirose, Ryo Tajima, Kento Hoshino, Yuka Miyake, Tsugumi Fukunaga, Toshiko Kato, Akari Endo, Hiroki Ito, Shingo Nakayama, Hideaki Hashimoto, Katsuya Ishiyama, Tomoyoshi Kimura, Takefumi Mori

**Affiliations:** 1 Division of Nephrology and Endocrinology, Tohoku Medical and Pharmaceutical University, Sendai 983-8536, Japan; tsato231@gmail.com (T.S.); hirose-t@med.tohoku.ac.jp (T.H.); ryo8812@gmail.com (R.T.); hoshi.is.5120@gmail.com (K.H.); yuka.miyake1025@gmail.com (Y.M.); pokethrush@yahoo.co.jp (T.F.); ren.s.hi.wth.4428@gmail.com (T.K.); akari-endo820@hosp.tohoku-mpu.ac.jp (A.E.); hito@tohoku-mpu.ac.jp (H.I.); nakayama-s@tohoku-mpu.ac.jp (S.N.); hide.h@tohoku-mpu.ac.jp (H.H.); ishiyama@tohoku-mpu.ac.jp (K.I.); tkimura@tohoku-mpu.ac.jp (T.K.); tmori@tohoku-mpu.ac.jp (T.M.); 2 Department of Diabetes and Metabolism, Tohoku University Graduate School of Medicine, Sendai 980-8575, Japan; 3 Department of Urology, Tohoku Medical and Pharmaceutical University, Sendai 983-8536, Japan; 4 Division of Integrative Renal Replacement Therapy, Tohoku Medical and Pharmaceutical University, Sendai 983-8536, Japan

**Keywords:** peritoneal dialysis, peritonitis, risk factor, subcutaneous fat

## Abstract

*Background and Objectives*: Peritoneal dialysis (PD)-related peritonitis is a critical problem. However, preoperative risk factors for PD-related peritonitis have not been established. Thus, we aimed to determine the preoperative risk factors for PD-related peritonitis. *Materials and Methods*: This is a single-center prospective observational study. All peritonitis episodes during the study period were recorded, and preoperative and intraoperative clinical parameters were compared between patients with and without peritonitis to examine risk factors for PD-related peritonitis. Furthermore, subcutaneous and abdominal fat volumes were evaluated using computed tomography. *Results*: Among a total of 118 patients, 24 patients developed peritonitis. The proportion of male patients (83% vs. 61%, *p* = 0.04), body mass index (25 vs. 22 kg/m^2^, *p* = 0.04), and subcutaneous fat area (120 vs. 102 cm^2^, *p* = 0.01) were significantly higher and the proportion of patients living with family members (75% vs. 94%, *p* = 0.02) was significantly lower in the peritonitis group than in the non-peritonitis group. There were no significant differences in age, operation method, surgeon experience, previous abdominal surgery, medical history of diabetic nephropathy, serum albumin level, and renal function between the two groups. *Conclusions*: Male patients with high subcutaneous fat who are living alone might be at higher risk of PD-related peritonitis. These characteristics might be useful in risk assessment and patient education before PD induction.

## 1. Introduction

Peritoneal dialysis (PD), an effective renal replacement therapy for patients with end-stage kidney disease (ESKD), has many advantages over hemodialysis [1,2,3]. However, several serious issues, including PD-related peritonitis, prevent its widespread use. PD-related peritonitis often leads to peritoneal dysfunction, catheter removal, transition to hemodialysis, progression to encapsulating peritoneal sclerosis, and death [4,5,6,7]. In Japan, the incidence of PD-related peritonitis was 0.23 patient-years in 1996, according to a retrospective study, and one episode per 42.8 patient-months in a recent multicenter study from 2005 to 2008 [7,8]. Several modifiable and non-modifiable clinical variables, such as male and female sex, high body mass index (BMI), diabetes mellitus, and low serum albumin levels, have been reported as risk factors for PD-related peritonitis [9,10,11,12,13,14,15,16,17,18,19,20,21,22]. However, the impact of these factors on the outcomes of patients with PD-related peritonitis have not been fully established.

Importantly, identification of preoperative risk factors for PD-related peritonitis is crucial to determine the timing of PD induction and to ensure its proper management. In the present study, we aimed to determine the impact of preoperative factors on PD-related peritonitis in a prospective cohort study of patients who were introduced to PD and followed at our institution. We compared patient background characteristics, including subcutaneous and intra-abdominal fat and total fat areas as well as subcutaneous fat thickness, using preoperative computed tomography (CT) and intraoperative parameters, such as operation method and surgeon experience, between patients with and without PD-related peritonitis. 

## 2. Materials and Methods

### 2.1. Study Design

This is a single-center prospective observational study including patients who were introduced to PD and followed for >6 months from January 2017 to February 2021 in Tohoku Medical and Pharmaceutical University, Japan. The inclusion criterion was patients aged ≥18 years. The exclusion criterion was patients with a follow-up duration of <6 months. The primary outcome was PD-related peritonitis. The secondary outcome was the characterization of identified microorganisms. 

A total of 118 eligible cases were included in the final analysis. All patients underwent PD catheter placement via open surgery using a stepwise initiation of PD as per the Moncrief and Popovich technique (SMAP) or direct method [23,24]. In the SMAP method, the catheter was implanted under the skin and withdrawn to create an exit site 3–6 months after the operation for catheter implantation. The selection of operation was based on the rate of decline in renal function. In general, the preparation for PD initiation was started in patients who reached stage 5 chronic kidney disease. The approach of choice was the SMAP method in patients with an estimated glomerular filtration rate of <10 mL/min/1.73 m^2^ for several months and the direct method in patients with unsuccessful conservative therapy who experienced rapid decline in renal function. All procedures were performed in a single operation in patients undergoing PD catheter replacement using the direct method.

### 2.2. Hypothesis

Diabetes mellitus and high BMI are reported as significant factors for PD-related peritonitis [8,9,13,14]. Therefore, we hypothesized that obesity would be a factor associated with PD-related peritonitis. In this study, subcutaneous, intra-abdominal, and total fat areas as well as subcutaneous fat thickness were evaluated using preoperative CT.

### 2.3. Data Collection and Definition

The diagnosis of PD-related peritonitis was based on the presence of two or more of the following findings: (1) abdominal pain, (2) cloudy effluent, (3) effluent white blood cell count >100/µL with at least 50% neutrophils, and (4) positive Gram staining or culture of the effluent [25]. Relapsing peritonitis, which was defined as an episode that occurred within 4 weeks after the completion of therapy for a prior episode with the same organism or sterile episode according to the International Society for Peritoneal Dialysis (ISPD) guidelines, was not included in the present study [25]. All episodes of peritonitis that occurred during the study period were recorded for analysis to prevent potential selection bias. 

The following preoperative clinical factors at the time of PD induction were included in the study: age, sex, BMI, primary renal disease (including diabetic nephropathy), previous abdominal surgery history, and PD duration. The intraoperative clinical factors included in the study were surgeon experience (instructor or not) and surgical method (direct or SMAP). In addition, we examined subcutaneous, intra-abdominal, and total fat areas as well as subcutaneous fat thickness using preoperative CT using the SYNAPSE VINCENT^®^ image analysis software (Fuji Film, Tokyo, Japan) to measure these parameters at the level of the umbilicus [26]. All images were analyzed by two researchers who were blinded to the clinical data.

The following laboratory parameters at the time of PD induction were included in the analysis: serum albumin (g/dL), hemoglobin (g/dL), white blood cell count (/μL), estimated glomerular filtration rate (mL/min/1.73 m^2^), total cholesterol (mg/dL), and triglycerides (mg/dL). The medical records were examined to collect data on the use of concomitant medications, such as oral active vitamin D and steroids, and to determine whether the patient was living alone or with family members at the time of PD induction.

### 2.4. Statistical Analysis

In the present study comparing patients who developed PD-related peritonitis (peritonitis group) to those who did not develop PD-related peritonitis (non-peritonitis group) during the study period, patient characteristics were described using medians, with ranges for continuous variables. The Shapiro–Wilk test was used to test the normality of the distribution. Intergroup differences in continuous variables were assessed using the Mann–Whitney U test, and categorical variables were assessed using the chi-squared or Fisher’s exact test. Multivariate analysis was performed using logistic regression. All statistical analyses were performed using the John Macintosh Project (JMP) statistical software version 12.2 (SAS Institute, Cary, NC, USA). *p* values of <0.05 were considered to indicate statistical significance.

## 3. Results

A total of 118 patients were enrolled in the present study. The clinical characteristics of the study patients are shown in Table 1. Briefly, 24 of the 118 patients developed peritonitis during the study period, and the median time to the first onset of PD peritonitis was 6 months (4–12). There were 20 male and four female patients in the PD peritonitis group and 57 male and 37 female patients in the non-peritonitis group. The proportion of male patients and the median BMI (83% vs. 61%, *p* = 0.04 and 25 vs. 22 kg/m^2^, *p* = 0.04, respectively) were significantly higher and the proportion of patients living with family members was significantly lower (75% vs. 94%, *p* = 0.02) in the peritonitis group than in the non-peritonitis group. However, there were no significant intergroup differences in age, operation method (direct or SMAP), surgeon experience, history of abdominal surgery, history of diabetic nephropathy, hypertension, hyperlipidemia, and coronary artery disease between the two groups.

The analyses of subcutaneous, intra-abdominal, and total fat areas as well as subcutaneous fat thickness via CT between the two groups are shown in Table 2. Remarkably, the subcutaneous fat area, subcutaneous fat thickness, and total fat area were significantly higher in the peritonitis group than in the non-peritonitis group (120 vs. 102 cm^2^, *p* = 0.01; 20 vs. 17 mm, *p* = 0.04; and 268 vs. 218 cm^2^, *p* = 0.02, respectively). In contrast, there was no significant intergroup difference in abdominal fat area between the two groups. Furthermore, there were no significant differences in serum albumin levels, renal function, lipid levels, white blood cell count, and hemoglobin level between the two groups.

We investigated the predictive factors for PD peritonitis in Table 3. Being male, subcutaneous, and living with family members were significant predictive factors for PD peritonitis [[odds ratio (OR), 3.25; *p* = 0.03 (univariate), OR, 4.12; *p* = 0.02 (multivariate)]; [OR, 1.01; *p* = 0.01 (univariate), OR, 1.01; *p*= 0.01 (multivariate)]; and [OR, 0.20; *p* = 0.02 (univariate), OR, 0.54; *p* = 0.42 (multivariate)], respectively].

The microorganisms and bacteria that caused PD-related peritonitis in the present study are shown in Table 4. Among the cultured bacterial isolates, the most common were Gram-positive bacteria (12 [46.1%] isolates), among which *Coagulase-negative Staphylococci* were the most frequent bacteria (4 [15.4%]). *Staphylococcus aureus* was present in two (7.7%) isolates, whereas Gram-negative bacteria were present in eight (30.8%) isolates. *Pseudomonas aeruginosa*, *Serratia*, and *Candida species* were also detected. Finally, culture-negative PD-related peritonitis was present in five (19.2%) patients.

## 4. Discussion

Although PD is an effective renal replacement therapy in patients with ESKD [1,2,3], peritonitis is one of the most crucial complications and a common cause of withdrawal from PD [4,5,6,7]. The PD-related peritonitis incidence of one episode per 59.0 patient-months is similar to that reported in a recent multicenter study in Japan (one episode per 42.8 patient-months) [7]. According to the 2016 ISPD guidelines, the incidence of PD-related peritonitis should not exceed 0.5 episodes per patient-year [27]. The incidence of 0.21 episodes per patient-year in the present study is in agreement with the ISPD recommendation. The identification of risk factors is important to predict and prevent the development of PD-related peritonitis. Previous studies reported several risk factors for PD-related peritonitis, such as male or female sex, high BMI, diabetes mellitus, older age, coronary artery disease, hypertension, hypoalbuminemia, and absence of vitamin D supplementation [9,10,11,12,13,14,15,16,17,18]. In the present study, we demonstrated that male sex, high BMI, high subcutaneous fat area, and living alone were preoperative risk factors for PD-related peritonitis.

In the present study, BMI was significantly higher in patients with PD-related peritonitis than in those without PD-related peritonitis. High BMI has been previously reported as a modifiable risk factor for peritonitis [9,13,14]. Furthermore, we demonstrated that subcutaneous and total fat areas were significantly higher in the peritonitis group than in the non-peritonitis group. The mechanism underlying the association between high BMI and peritonitis has not yet been clarified. However, several possibilities might explain the high subcutaneous fat area observed in patients with PD-related peritonitis. First, high subcutaneous fat reflects that the PD catheter has to pass through more subcutaneous tissue, which can potentially increase the risk of catheter infection. Second, subcutaneous fat may cause wrinkles and sagging in the abdomen or may increase catheter mobility, leading to catheter loosening, which may hinder PD tube management necessary to preserve a clean catheter. The present study results suggest that assessing subcutaneous fat using preoperative CT at the time of PD induction might be useful in risk assessment for peritonitis.

Interestingly, we found that PD-related peritonitis was significantly higher among the patients not cohabitating with family members. Although living far from PD management facilities or clinics was reported as a risk factor for PD-related peritonitis [28,29], to the best of our knowledge, this is the first report examining the impact of family support through living together as a risk factor for PD-related peritonitis. A study reported that patients changed the standard catheter maintenance methods and became noncompliant with aseptic manipulation approximately 6 months after PD initiation [30]. Assisted PD by a family member did not predict peritonitis when compared with self-care PD, whereas assisted PD was associated with a 2.6-fold increased risk of peritonitis [31]. Assisted PD and employment status were associated with PD training duration [32]. This previous report suggested that inconsistent homecare aid and inadequate training increase the risk of PD-related peritonitis. Although we were not able to confirm whether the family members living with the patient checked or performed the procedure in the present study cohort, assistance by family members might have led to the observation of the correct technique and management. Home visits and reeducation by nurse have been suggested to contribute to a reduction in the risk of PD-related peritonitis [33,34], and guidelines and educational sources for patients who live alone should be carefully considered.

Gram-positive bacteria were the most commonly isolated bacteria in patients with PD-related peritonitis, and *Coagulase-negative staphylococci* were the most common bacteria (Table 4), a finding that is in strong agreement with other studies in a Japanese cohort [8,21]. Importantly, in the present study, the rate of negative cultures was approximately 20%, in accordance with the standard ISPD guidelines which state that the rate of culture-negative peritonitis should not be greater than 20% [27]. Also, the proportion of Gram-negative bacteria with PD-related peritonitis has been reported to be increasing recently (17–32%), and our result is consistent with this trend [35,36].

The present study has several limitations that warrant acknowledgment. First, this was a single-center prospective observational study with a relatively small sample size, and prospective multicenter studies comprising larger patient cohorts are necessary for more definitive evidence. Second, it was unclear with whom or under whose supervision PD was performed in each patient. Data on the use of home nursing and employment status, especially of single male patients, were not collected. However, the current study findings suggested that male sex, high subcutaneous fat, and living alone were significant risk factors of PD-related peritonitis. These findings might be useful during the consideration of PD indications and for the implementation of education and guidelines for patients undergoing PD. 

## 5. Conclusions

Male sex, high subcutaneous fat, and living alone were significantly associated with PD-related peritonitis in this study. The determination of subcutaneous fat area using preoperative CT at the time of PD induction may be useful for the risk assessment of peritonitis, and obese single males might be at a higher risk of peritonitis.

## Figures and Tables

**Table 1:** Comparison of background characteristics between patients with and without peritoneal dialysis-related peritonitis.

Group	With PD-Related Peritonitis (*n* = 24)	Without PD-Related Peritonitis (*n* = 94)	*p* Value
Age (years)	73 (54–85)	77 (66–83)	0.18
Sex (male, %)	20 (83%)	57 (61%)	0.04
Body mass index (kg/m^2^)	25 (21–28)	22 (20–25)	0.04
Operation (SMAP, %)	9 (38%)	49 (52%)	0.20
Surgeons (instructor, %)	14 (58%)	61 (65%)	0.55
PD duration (months)	18 (8–30)	16 (8–22)	0.43
Previous abdominal surgery (*n*, %)	7 (29%)	21 (23%)	0.50
Diabetic nephropathy (*n*, %)	12 (50%)	47 (50%)	0.50
Living with family members (*n*, %)	18 (75%)	88 (94%)	0.02
Use of steroids (*n*, %)	1 (4%)	7 (7%)	0.57
Use of oral vitamin D (*n,* %)	12 (50%)	55 (59%)	0.45
Hypertension (*n*, %)	23 (96%)	82 (87%)	0.23
Hyperlipidemia (*n*, %)	8 (33%)	42 (45%)	0.32
Coronary artery disease (*n*, %)	3 (13%)	21 (22%)	0.29

Data are presented as medians (first quartile–third quartile) or count (%). Abbreviations: PD, peritoneal dialysis; SMAP, stepwise initiation of peritoneal dialysis using Moncrief and Popovich.

**Table 2:** Comparison of preoperative CT findings and laboratory parameters between patients with and without peritoneal dialysis-related peritonitis.

Group	With PD-Related Peritonitis (*n* = 24)	Without PD-Related Peritonitis (*n* = 94)	*p* Value
Subcutaneous fat area (cm^2^)	120 (83–227)	102 (57–146)	0.01
Intra-abdominal fat area (cm^2^)	122 (92–182)	116 (74–148)	0.10
Total fat area (cm^2^)	268 (187–374)	218 (150–283)	0.02
Subcutaneous fat thickness (mm)	20 (12–28)	17 (11–23)	0.04
eGFR (mL/min/1.73 m^2^)	10 (7–13)	10 (7–12)	0.94
BUN (mg/dL)	64 (42–76)	56 (45–73)	0.66
Total protein (g/dL)	6.4 (6.0–6.7)	6.3 (5.6–6.9)	0.70
Serum albumin (g/dL)	3.5 (2.8–3.6)	3.2 (2.8–3.6)	0.58
Total cholesterol (mg/dL)	147 (130–187)	157 (128–184)	0.56
Triglycerides (mg/dL)	123 (95–167)	116 (87–155)	0.74
White blood cell (/μL)	5900 (4900–6800)	5800 (4800–7800)	0.44
Hemoglobin (g/dL)	10.7 (9.8–12.1)	10.3 (9.0–11.2)	0.20

Data are presented as medians (first quartile–third quartile) or count (%). Abbreviations: BUN, blood urea nitrogen; eGFR; estimated glomerular filtration rate; PD, peritoneal dialysis.

**Table 3:** Predictive factors for peritoneal dialysis-related peritonitis.

Variable	Univariate			Multivariate		
	Odds Ratio	95%CI	*p* Value	Odds Ratio	95%CI	*p* Value
Age (years)	0.98	0.95–1.01	0.18			
Sex (male, %)	3.25	1.12–11.83	0.03	4.12	1.22–18.20	0.02
Body mass index (kg/m^2^)	1.10	1.01–1.22	0.04			
Subcutaneous fat area (cm^2^)	1.01	1.00–1.01	0.01	1.01	1.00–1.01	0.01
Intra-abdominal fat area (cm^2^)	1.01	0.99–1.01	0.10			
Living with family members	0.20	0.06–0.72	0.02	0.54	0.13–2.48	0.42

Abbreviations: CI, confidence interval.

**Table 4:** Characteristics of identified microorganisms and bacteria.

	Number of Isolates (*n* = 26) *	% of Total **
Gram-positive bacteria	12	46.1
*C* *oagulase-negative staphylococci*	4	15.4
*Staphylococcus aureus*	2	7.7
*Streptococcus*	2	7.7
*Enterococcus*	1	3.8
*Corynebacterium*	1	3.8
Other	2	7.7
Gram-negative bacteria	8	30.8
*Escherichia coli*	2	7.7
*Klebsiella species*	2	7.7
*Pseudomonas*	1	3.8
*Serratia*	2	7.7
Other	1	3.8
*Candida species*	1	3.8
Culture-negative	5	19.2

* The total number did not match the number of peritonitis patients (*n* = 24), because more than one organism was detected in some cases. ** Sum does not equal 100% due to rounding.

## Data Availability

Detailed data presented in the study are available on request from corresponding author.
